# Identification of a Conserved B-Cell Epitope on Duck Hepatitis A Type 1 Virus VP1 Protein

**DOI:** 10.1371/journal.pone.0118041

**Published:** 2015-02-23

**Authors:** Xiaoying Wu, Xiaojun Li, Qingshan Zhang, Shaozhou Wulin, Xiaofei Bai, Tingting Zhang, Yue Wang, Ming Liu, Yun Zhang

**Affiliations:** State Key Laboratory of Veterinary Biotechnology, Harbin Veterinary Research Institute of Chinese Academy of Agricultural Sciences, Harbin, 150001, P. R. China; Chinese Academy of Medical Sciences, CHINA

## Abstract

**Background:**

The VP1 protein of duck hepatitis A virus (DHAV) is a major structural protein that induces neutralizing antibodies in ducks; however, B-cell epitopes on the VP1 protein of duck hepatitis A genotype 1 virus (DHAV-1) have not been characterized.

**Methods and Results:**

To characterize B-cell epitopes on VP1, we used the monoclonal antibody (mAb) 2D10 against *Escherichia coli*-expressed VP1 of DHAV-1. *In vitro*, mAb 2D10 neutralized DHAV-1 virus. By using an array of overlapping 12-mer peptides, we found that mAb 2D10 recognized phages displaying peptides with the consensus motif LPAPTS. Sequence alignment showed that the epitope ^173^LPAPTS^178^ is highly conserved among the DHAV-1 genotypes. Moreover, the six amino acid peptide LPAPTS was proven to be the minimal unit of the epitope with maximal binding activity to mAb 2D10. DHAV-1–positive duck serum reacted with the epitope in dot blotting assay, revealing the importance of the six amino acids of the epitope for antibody-epitope binding. Competitive inhibition assays of mAb 2D10 binding to synthetic LPAPTS peptides and truncated VP1 protein fragments, detected by Western blotting, also verify that LPAPTS was the VP1 epitope.

**Conclusions and Significance:**

We identified LPAPTS as a VP1-specific linear B-cell epitope recognized by the neutralizing mAb 2D10. Our findings have potential applications in the development of diagnostic techniques and epitope-based marker vaccines against DHAV-1.

## Introduction

Duck hepatitis A virus (DHAV), formally known as duck hepatitis virus type 1 (DHV-1), is a member of the genus Avihepatovirus in the family *Picornaviridae*; it is genetically divided into three serotypes: the original DHAV-1, a serotype that was isolated in Taiwan (DHAV-2), and a serotype that was isolated in South Korea and China (DHAV-3) [2–6]. There is no cross-neutralization between DHAV-2 or DHAV-3 and DHAV-1 [4, 6].

DHAV-1 disease is a fatal, rapidly spreading viral infection of young ducklings that is characterized primarily by hepatitis [7]. DHAV-1 disease has spread worldwide and continues to be a threat to duck farms because of the high mortality associated with the disease. Mortality in the field often exceeds 50% and can reach 95% [8]. The hepatic clinical signs caused by DHAV-1, DHAV-2, and DHAV-3 are difficult to differentiate [2,3,5].

Among the picornaviruses, the most distinctive divergence is usually located in VP1 [9,10]. VP1 is the most external and immunodominant of the picornavirus capsid proteins, potentially containing B and T cell epitopes that could induce protective neutralizing antibodies [11]. The VP1 of foot and mouth disease virus (FMDV), coxsackievirus A9 (CAV-9), echovirus (EV), human parechovirus 1 (HPeV-1), and human parechovirus 2 (HPeV-2) has an RGD motif, which is located in the G-H loop of FMDV VP1, and in the C terminus of CAV-9, EV, HPeV-1, and HPeV-2 VP1 [9, 12–17]. Like that of HPeV3 [11], the VP1 of DHAV-1, DHAV-2, and DHAV-3 lacks an RGD motif [1, 2, 4, 6].

In this study, we used a mAb against VP1 protein (2D10) to screen a phage-display random 12-mer peptide library for the linear B-cell epitope of VP1. This report thus documents the first epitope analysis of the VP1 protein of DHAV-1. By furthering our understanding of the antigenic structure of VP1, our findings will facilitate the specific serologic diagnosis of DHAV-1 infection and will contribute to the rational design of effective vaccines.

## Materials and Methods

### Cells, Viruses, Sera, and the VP1-specific mAb 2D10

DHAV-1 HP-1 and DHAV-3 JT strains were grown on duck embryo fibroblasts cells (DEF) or duck embryonated eggs as described previously [5, 18, 19]. Duck sera against DHAV-1 HP-1 and sera from uninfected healthy ducks were prepared as described previously [18]. The mAb 2D10 was prepared as described elsewhere [19].

### Ethics Statement

Care of laboratory animals and animal experimentation were performed in accordance with animal ethics guidelines and approved protocols. All animal studies were approved by the Animal Ethics Committee of the Harbin Veterinary Research Institute of Chinese Academy of Agricultural Sciences.

### Indirect Immunofluorescence Assay

Detection of the VP1 protein of DHAV-1 or DHAV-3 by means of an immunofluorescence assay was performed according as previously described with modifications [19]. Briefly, confluent DEF cells were infected with DHAV-1 HP-1 or DHAV-3 JT virus (10 M.O.I.) and then incubated at 37°C for 72 h. The cells were fixed with cold methanol for 10 min and then probed with mAb 2D10 or uninfected mouse serum (as a control) for 1 h at 37°C. Then, 50 μL/well of FITC-conjugated goat anti-mouse IgG (KPL, MD, USA) at a 1:400 dilution was added and the cells were incubated for 1 h at 37°C. To confirm the location of the VP1 protein, the cells were then stained with DAPI as described previously [20]. The stained cells were viewed by using a Zeiss Axioplan-2 or a confocal LSM 700 (Carl Zeiss) fluorescence microscope.

### *In Vitro* Neutralization Assay

Neutralizing antibody titers of mAb 2D10 were detected by using a virus-based neutralization assay as previously described [18]. Briefly, 100 μL of serial diluted mAb (initial dilution, 1:10; 2-fold dilutions to 1:320) was incubated with 100 μL 1×10^2.5^ TCID_50_ of DHAV-1 HP-1 for 2 h at 37°C. The virus-mAb mixture (200 μL) was then transferred onto a monolayer of DEF cells in a 96-well plate (triplicate wells). Sera from uninfected healthy mice (diluted in phosphate-buffered saline, PBS) and uninfected DEF cells served as controls. Cells were observed daily for cytopathic effects (CPE) for 7 days. Neutralization titers were read as the highest mAb dilution that protected >95% of the cells from CPE.

### Epitope Mapping

The Ph.D-12 Phage Display Peptide Library Kit (New England BioLabs Inc) was used in this study. The mAb 2D10 was purified from mice ascites fluid by using Protein G Agorose (Invitrogen, Carlsbad, CA, USA) according to the manufacturer’s instructions. Three successive rounds of biopanning were carried out in accordance with the manufacturer’s instructions. Briefly, each well of a 96-well plate was coated with 10 μg/mL of mAb 2D10 in coating buffer overnight at 4°C, and then blocked with blocking buffer for 2 h at 4°C. The phage library was added to the plate and incubated for 1 h at room temperature. The unbound phages were removed by successive washings with TBS buffer containing gradually increased concentrations (0.1%, 0.3%, and 0.5%) of Tween-20, and the bound phages were eluted by 0.2 M glycine-HCl containing 1 mg/mL BSA and immediately neutralized with 1 M Tris-HCl. The eluted phages were amplified and titered on LB/IPTG/Xgal plates for the subsequent rounds of selection. The ratio of output to input was calculated as follows: titer of the amplified output phages/titer of the input phages (1.5×10^11^)×100%.

### Phage ELISA and Sequencing of DNA Inserts Displayed by Phage Clones

After three rounds of biopanning, 15 individual phage clones were selected for target binding in the ELISA as described in manufacturer’s instructions. Briefly, 96-well plates were coated with 100 ng of purified mAb 2D10, or anti-porcine IFN-c mAb (Sigma, St Louis, MO, USA) as negative a control overnight at 4°C. The coated wells were blocked for 2 h at room temperature and then the phages (10^10^ pfu/100 μL/well) diluted in blocking solution were added. The plates were incubated for 1 h at room temperature and were then washed ten times with TBST. Bound phages were subjected to reaction with horseradish peroxidase (HRP)-conjugated sheep anti-M13 antibody (Pharmacia, Piscataway, NY, USA), followed by color development with the substrate solution containing o-phenylenediamine (OPD). The positive phage clones were sequenced with the sequencing primer as described in the manufacturer’s instructions.

### Peptide Design and Synthesis

After bioinformatics analysis of selected clones, several peptide sequences were designed and synthesized (with purity >95%) by GenScript China Inc. P11 represents residues LPAPTS; P11ΔL, p11ΔS, p11ΔLP, and p11ΔTS represent residues PAPTS, LPAPT, APTS, and LPAP, respectively.

### Immunological Analysis of Synthetic Peptides

Dot blotting was performed by spotting a synthesized peptide solution onto a nitrocellulose membrane. Approximately 1 μg of each synthesized peptide diluted with TNE buffer was spotted onto the nitrocellulose membrane. The membrane was then incubated with mAb 2D10 (diluted 1:2,000 in PBS) or anti-porcine IFN-c mAb (negative control) and duck-positive anti-DHAV-1 sera or healthy uninfected duck sera (negative control) (diluted 1:100 in PBS) at 37°C for 1 h. After being washed three times with PBST, the membranes were probed with a 1:500 dilution of HRP-conjugated goat anti-mouse IgG (KPL, MD, USA) or goat anti-duck IgG (KPL, MD, USA) at 37°C for 1 h, respectively.

### Competitive Inhibition Binding Assay of mAb 2D10 to Synthetic Peptide

To test for synthetic peptide inhibition of mAb 2D10 binding to VP1, 100 μL of VP1 antigen (10μg/mL) was coated onto 96-well plates (at 4°C overnight). Plates were then blocked as described previously. The synthetic peptide LPAPTS (final peptide concentrations 0, 5, 10, 20, 40, 80, and 160 μg/ml) or an unrelated control peptide (^19^YIRTPACWD^27^, from duck reovirus σB protein. [21]) was mixed with blocking mAb 2D10 (0.2 μg/ml diluted in PBST) and incubated at room temperature for 45 min; these peptide/antibody mixtures were then added to the VP1 antigen-coated 96-well plates and incubated at room temperature for 1 h. After the plate was washed with PBST, HRP-conjugated goat anti-mouse IgG was added and binding was assessed.

### Expression of Consecutive Truncated VP1 Protein Fragments and Western Blotting

Primers (S1 Table) specific for the VP1_p1–180_, VP1_p1–172_, VP1_p172–238_, VP1_p179–238_, and VP1_p173–178_ fragments were designed as described previously [18, 21]. The VP1_p1–180_ and VP1_p1–172_ fragment genes were cloned into pET30a (Novagen, Madison, WI, USA), and the VP1_p172–238_, VP1_p179–238_, and VP1_p173–178_ deletion genes were cloned into pGEX6p-1 (GE Healthcare) as described previously [21]. The expressed truncated VP1 fragments, which were purified by using the Ni-NTA kit (Qiagen, Valencia, CA) and GST Purification Kit (TaKaRa, Dalian, China), were subjected to 10% SDS-PAGE and transferred to nitrocellulose membranes. The membranes were probed with mAb 2D10 followed by a secondary HRP-conjugated goat anti-mouse antibody (KPL, MD, USA).

### Sequence Analysis

To assess the conservation of the epitope among the DHAV viruses, we performed sequence alignment of the corresponding epitope regions on the VP1 proteins of seven DHAV-1 strains (Table 1), two DHAV-3 strains, and one DHAV-2 strain by using the DNASTAR Lasergene program (DNASTAR Inc., Madison, WI, USA).

**Table 1:** Virus strains used in VP1 sequence analyses and their accession numbers in GenBank.

Species	Strain	Accession no.	Isolation
Duck hepatitis A virus (DHAV-1)	HP	EF151312	China
Duck hepatitis A virus (DHAV-1)	E53	EF151313	China
Duck hepatitis A virus (DHAV-1)	DRL-62	DQ219396	America
Duck hepatitis A virus (DHAV-1)	R85952	DQ226541	America
Duck hepatitis A virus (DHAV-1)	H	DQ249300	UK
Duck hepatitis A virus (DHAV-1)	5886	DQ249301	America
Duck hepatitis A virus (DHAV-1)	03D	DQ249299	Taiwan
Duck hepatitis A virus (DHAV-2)	90D	EF067924	Taiwan
Duck hepatitis A virus (DHAV-3)	AP-4009	DQ256133	Korea
Duck hepatitis A virus (DHAV-3)	JT	JF835025	China

## Results

### Characterization and Neutralization Analysis of mAb 2D10

**Fig 1:**
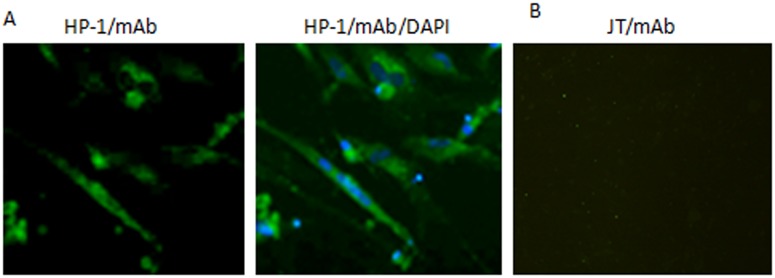
DHAV-1 HP-1- and DHAV-3 JT-infected DEF cells detected with mAb 2D10 by IFA. (A) DHAV-1 HP-1-infected DEF cells; Infected cells were stained with mAb 2D10 and then with an FITC-conjugated goat anti-mouse antibody, and finally with DAPI. Stained cells were visualized by means of fluorescence microscopy. (magnification 300×). (B) JT-infected DEF or uninfected DEF cells detected by mAb 2D10 (negative control).

The immunofluorescence assay revealed that mAb 2D10 reacted with DHAV-1 HP-1-infected DEF cells (Fig. 1A), but not with DHAV-3 JT- infected or uninfected DEF cells (Fig. 1B). Green fluorescence was visualized in DHAV-1 HP-1-infected cells, indicating that mAb 2D10 detected the native-form of the VP1 protein in the DHAV-1 HP-1 infected cells. DAPI staining indicated that VP1 was mainly located in the cytoplasm of the infected cells (Fig. 1A), although some VP1 were detected in the nucleus, which is consistent with a previous report on the VP1 protein of FMDV [22]. The neutralizing activity of mAb 2D10 was then determined by use of an *in vitro* neutralization assay on DEF cells; mAb 2D10 neutralized the DHAV-1 HP1 virus with a neutralization titer (NT_50_) of 40.

### Phage Enrichment by Biopanning

To determine the epitope recognized by mAb 2D10, bio-panning of a phage display 12-mer random peptide library was performed with the purified mAb. After three rounds of bio-panning, enrichment of phages bound to the mAb was obtained. The output to input ratios of the three rounds of bio-panning were 0.00009%, 0.037% and 0.78%.

### Epitope Prediction

**Fig 2:**
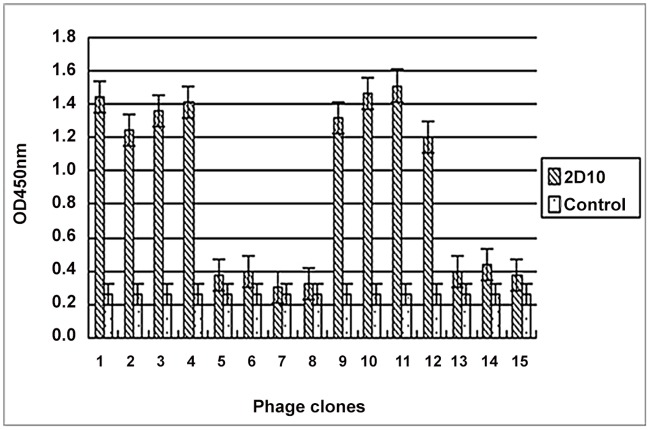
Detection of selected phages for antibody binding by Phage ELISA. After three rounds of biopanning, 15 selected phage clones were detected by mAb 2D10 or an anti-porcine IFN-c mAb (negative control). Three independent assays were performed for each selected phage.

Fifteen phage clones were selected for their reactivity with mAb 2D10 after three rounds of bio-panning. These selected clones were further evaluated in a Phage ELISA. As shown in Fig. 2, eight phage clones (p1–p4 and p9–p12) showed specific reactivity with 2D10 (OD450 nm. ≥1.20) but did not react with anti-porcine IFN-c mAb (OD450 nm, ˂0.33). The other 7 phage (p5–p8 and p13–p15) showed less reactivity with 2D10 (OD450 nm, OD˂0.55). The eight phage clones with high OD values were sequenced and found to contain the consensus sequence LPAPTS, which is identical to the sequence ^173^LPAPTS^178^ of the VP1 protein of DHAV-1 HP-1 (Table 2).

**Table 2:** Peptides sequences of eight phage clones and their consensus sequences.

Phage clone	Phage sequences											
1	—	S	D	S	**L**	**P**	T	**P**	**T**	**S**	N	T
2	—	S	M	L	T	A	P	**P**	**T**	**S**	R	D
3	H	S	Y	P	T	**P**	—	R	**T**	**S**	S	G
4	V	D	E	Y	G	T	**A**	L	**T**	**S**	—	—
9	S	H	T	Q	D	**P**	**A**	**P**	**T**	**S**	N	V
10	—	T	Q	M	**L**	**P**	**A**	**P**	D	L	P	S
11	A	T	S	T	Q	**P**	**A**	—	**T**	**S**	N	T
12	—	—	N	T	**L**	**P**	**A**	N	V	P	S	N
Consensus					**L**	**P**	**A**	**P**	**T**	**S**		
HP-1	**L**	**F**	**F**	**P**	**L**	**P**	**A**	**P**	**T**	**S**	**T**	**T**

Consensus sequences are shown underlined.

### Competitive Inhibition of Synthetic Peptide LPAPTS Binding to mAb 2D10

**Fig 3:**
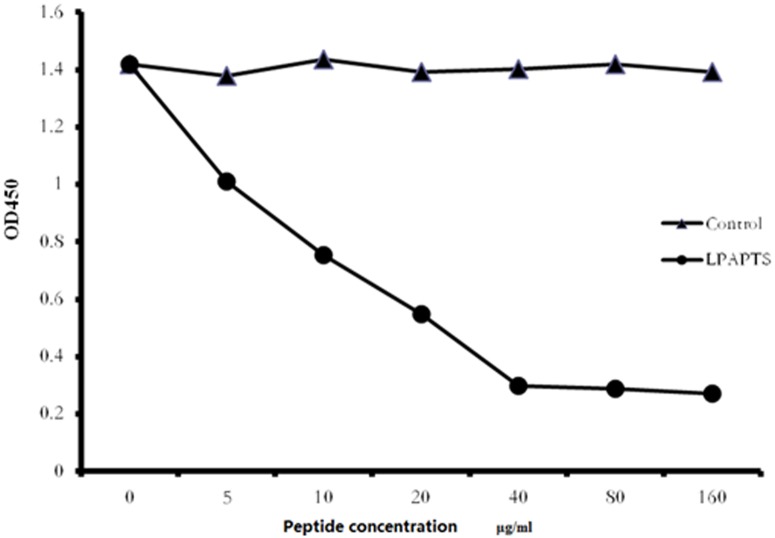
Competitive inhibition of synthetic peptide LPAPTS binding to mAb 2D10. A competitive ELISA was performed using the antigen peptide LPAPTS as the competitor for the VP1 protein. Values represent three independent experiments with triplicates for each experiment (*p* < 0.05).

To confirm that peptide LPAPTS of VP1 is the epitope of 2D10, competitive binding assays were performed. These assays showed that the reactivity of mAb 2D10 with VP1 protein was inhibited markedly by the synthetic antigen peptide LPAPTS in a dose-dependent manner (*p* < 0.05, Fig. 3). The control peptide (^19^YIRTPACWD^27^, from duck reovirus σB protein) showed no inhibition.

### Precise Epitope Defining

**Fig 4:**
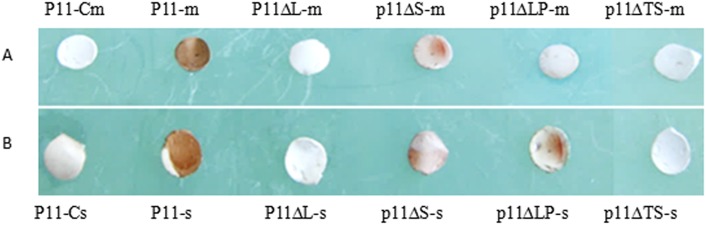
Dot blotting assay of mAb 2D10 and duck anti-DHAV-1 sera to synthesized peptides. (A) P11-m, P11ΔL-m, p11ΔS-m, p11ΔLP-m, and p11ΔTS-m represent the results for peptides LPAPTS, PAPTS, LPAPT, APTS, and LPAP reacted with mAb 2D10, respectively. P11-Cm represents the result for P11 reacted with mAb anti-porcine IFN-c (negative control). (B) P11-s, P11ΔL-s, p11ΔS-s, p11ΔLP-s, and p11ΔTS-s represent the results for peptides LPAPTS, PAPTS, LPAPT, APTS, and LPAP reacted with duck anti-DHAV-1 sera. P11-Cs represents the result for P11 reacted with uninfected duck sera (negative control).

To verify that the identified motif represented the epitope recognized by mAb 2D10 and duck anti-DHAV-1 sera, peptides representing the motif LPAPTS were synthesized. Dot blot analysis showed that the peptide LPAPTS was recognized by mAb 2D10 and duck anti-DHAV-1 sera (Fig. 4), but did not reacted with mAb anti-porcine IFN-c or healthy duck sera, indicating that the motif represented a linear B-cell epitope of the VP1 protein of DHAV-1. To define the epitope precisely, C- and N-terminal deletion mutants of the LPAPTS motif were synthesized. We found that only the full-length LPAPTS peptide was recognized by mAb 2D10 or duck anti-DHAV-1 sera. Removal of one or two amino acids at either the N- or C- terminus of the peptides abolished antibody binding (Fig. 4), indicating that the peptide LPAPTS represented the minimal requirement for the reactivity of the epitope with mAb 2D10.

### Epitope Identification by Western Blotting

**Fig 5:**
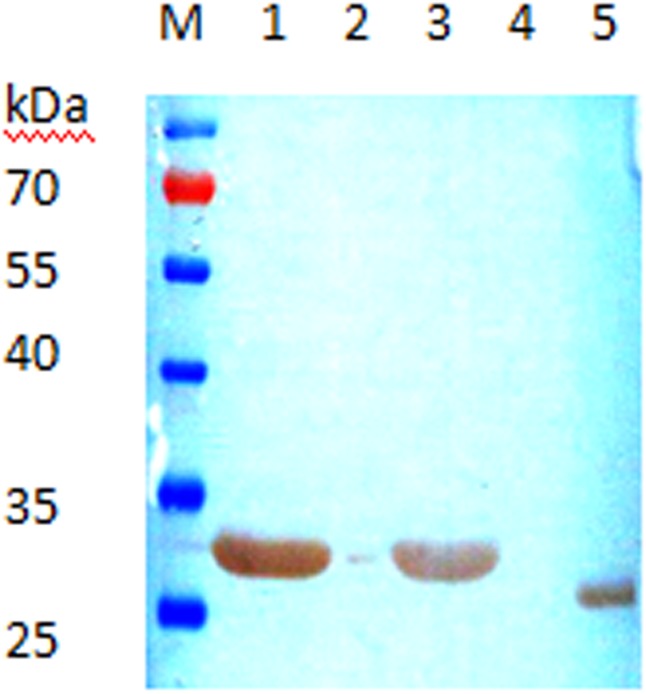
Identification of the VP1 epitope from the reactivity of mAb 2D10 with purified VP1-deletions by means of Western blotting. Lane 1, VP1_P 1–180_; lane 2, VP1_P 1–172_; lane 3, VP1 _P 173–238;_ lane 4, VP1_P 179–238_; and lane 5, VP1_P 173–178_.

To further define LPAPTS as the epitope of the VP1 protein, we analyzed the immunoreactivities of a series of truncated VP1 fragments to mAb 2D10 by use of Western blotting. The VP1_P1–180_ (about 33.7 kDa, including the 6×His tag) (Fig. 5, lane 1), VP1_P172–238_ (about 33.9 kDa, including the GST tag) (Fig. 5, lane 3), and VP1_P173–178_ (about 26.6kDa, including the GST tag) (Fig. 5, lane 5) fragments were recognized by mAbs 2D10, whereas VP1_P1–172_ (about 32.9 kDa, including the 6×His tag) (Fig. 5, lane 2) and VP1_P 179–238_ (33.1 kDa, including the GST tag) (Fig. 5, lane 4) were not recognized by mAb 2D10, suggesting that amino acids 173–178 of VP1 may represent the dominant antigen region of VP1. The VP1_P173–178_ fragment was recognized by mAb 2D10, confirming that ^173^LPAPTS^178^ was the exact epitope of the VP1 protein.

### Epitope LPAPTS is Highly Conserved among DHAV-1 Strains

**Fig 6:**
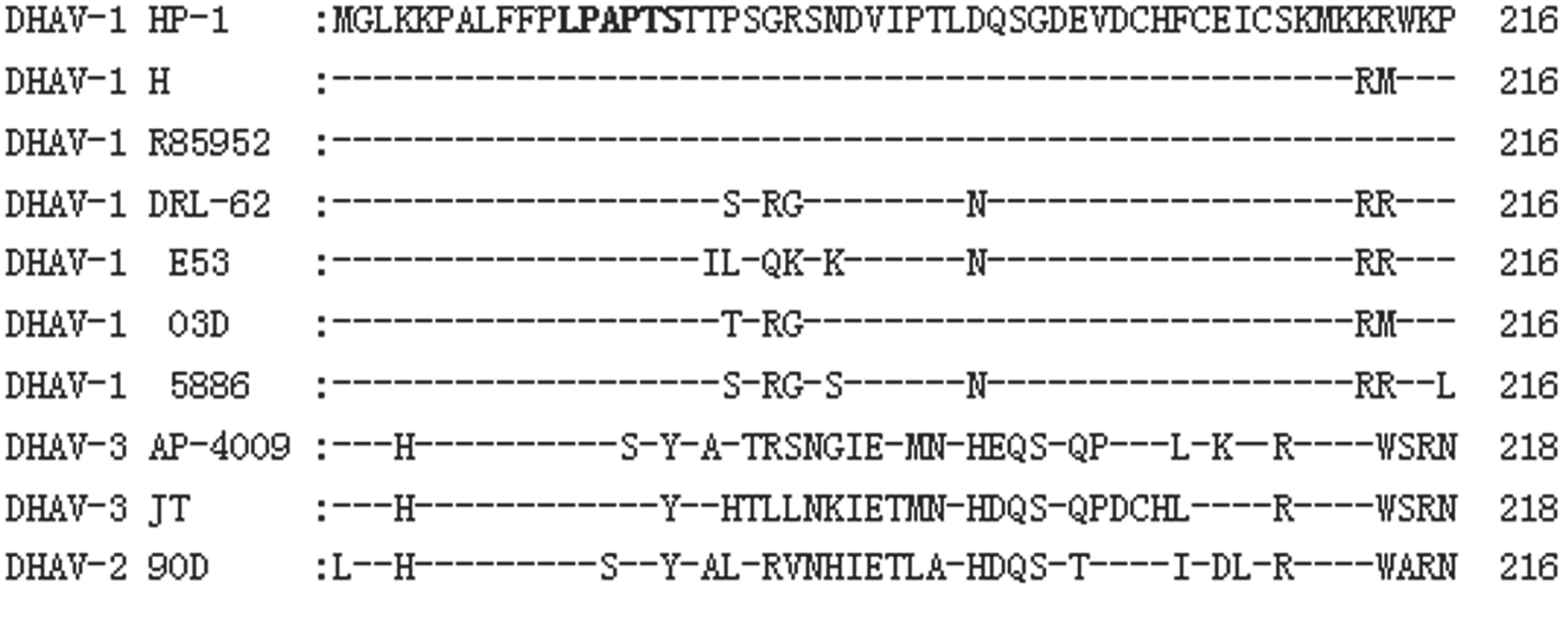
Sequence alignment of 10 DHAV strains around the epitope-coding region of the VP1 protein. Amino acid positions for each individual sequence are numbered on the right. DHAV-1 HP-1 strain sequences are shown at the top; dashes indicate identical amino acids. The identified epitope is in bold.

To investigate the conservation of the LPAPTS epitope, we aligned the VP1 partial sequence, including the epitope region identified in this study, with other DHAV-1 sequences available in GenBank (Table 2). The alignment results showed that all of the amino acids in the motif region were identical among DHAV-1 strains (Fig. 6), but differed from the amino acids in the corresponding regions of DHAV-2 and DHAV-3, indicating that the motif represented a conserved epitope on the VP1 protein of DHAV-1.

## Discussion

The VP1 protein of DHAV-1 is associated with virus neutralization and is, therefore, a major candidate antigen for vaccine development and disease serological diagnosis [18, 23]. Epitope mapping using monoclonal antibodies has become a powerful means to study protein structure and has proved to be an effective strategy for vaccine development and disease diagnosis [24–26]. To our knowledge, there have been no previous reports of the linear epitope mapping of the VP1 of DHAV.

In this investigation, we first characterized mAb 2D10 against VP1 of DHAV-1. IFA showed that mAb 2D10 recognized DHAV-1 virus-infected DEF cells but not DHAV-3 virus-infected cells, confirming previous reports that DHAV-1 shows no cross reactivity with DHAV-3 [4, 5, 7, 18]. IFA also showed that the VP1 protein was located mainly in the cytoplasm of DHAV-1-infected DEF cells, but DAPI staining revealed that some VP1 protein was also distributed in the nucleus of infected cells, which is consistent with the location of VP1 in FMDV-infected cells [22]. Most proteins enter the nucleus via nuclear localization signals (NLSs). NLSs have been shown to contain a continuous motif of basic amino acid residues [20, 27, 28]; When we scanned the VP1 protein sequence, we noticed a cluster of basic amino acids (^189^ KMKKRWKPR ^197^) (basic amino acids underlined) (S2 Table) at the C-terminal of the VP1 protein, which may represent an NLS. Whether this basic motif serves some nuclear import function should be evaluated. The linear epitope LPAPTS recognized by mAb 2D10 was identified by using a random phage display peptide library. Sequence analysis confirms that the epitope sequence was identical to ^173^LPAPTS^178^ of the VP1 protein of DHAV-1. Alignment of DHAV-1, DHAV-2, and DHAV-3 virus strain sequences demonstrated that this epitope sequence was highly conserved among DHAV-1 viruses, but not among DHAV-2 and DHAV-3 viruses, indicating that it is a DHAV-1 type-specific epitope. This finding supports previous reports that there is no cross reactivity between DHAV-1 and DHAV-2 or DHAV-1 and DHAV-3 [2, 4, 5, 7, 18]. Dot blotting assays with mAb 2D10 and N- or C- terminal deletion mutants of the epitope demonstrated that the peptide LPAPTS was the minimal unit required for 2D10 recognition (i.e. maximal binding to mAb 2D10). The peptide LPAPTS was also recognized by DHAV-1-positive duck serum, suggesting the importance of these six amino acids of the epitope in antibody-epitope binding reactivity. Moreover, the competitive inhibition assay of mAb 2D10 binding to synthetic LPAPTS and to truncated VP1 protein fragments, as detected by Western blotting, verify that LPAPTS was the epitope of the VP1 protein. The location of the epitope ^173^LPAPTS^178^ in the C-terminus of VP1 of DHAV-1 is consistent with recent studies of FMDV, CAV-9, EV-9, HPeV-1 and HPeV-2 viruses [9, 11–17]. Further experiments are necessary to confirm the functional role of this epitope.

## Conclusions

In summary, a highly conserved neutralizing linear B-cell epitope on the VP1 protein of DHAV-1 was identified. This conserved epitope may have potential use in the development of DHAV-1–specific diagnostic assays and epitope-based marker vaccines.

## Supporting Information

S1 Table Primers for truncated VP1 protein fragments. (TIF)

S2 Table DHAV-1 HP-1 strain VP1 amino acids sequence. (TIF)
