# Supplementary figures and images for: Identification of a Conserved B-Cell Epitope on Duck Hepatitis A Type 1 Virus VP1 Protein
Source: PLoS One. 2015 Feb 23;10(2):e0118041. doi: 10.1371/journal.pone.0118041 (PMC4337900; doi:10.1371/journal.pone.0118041)

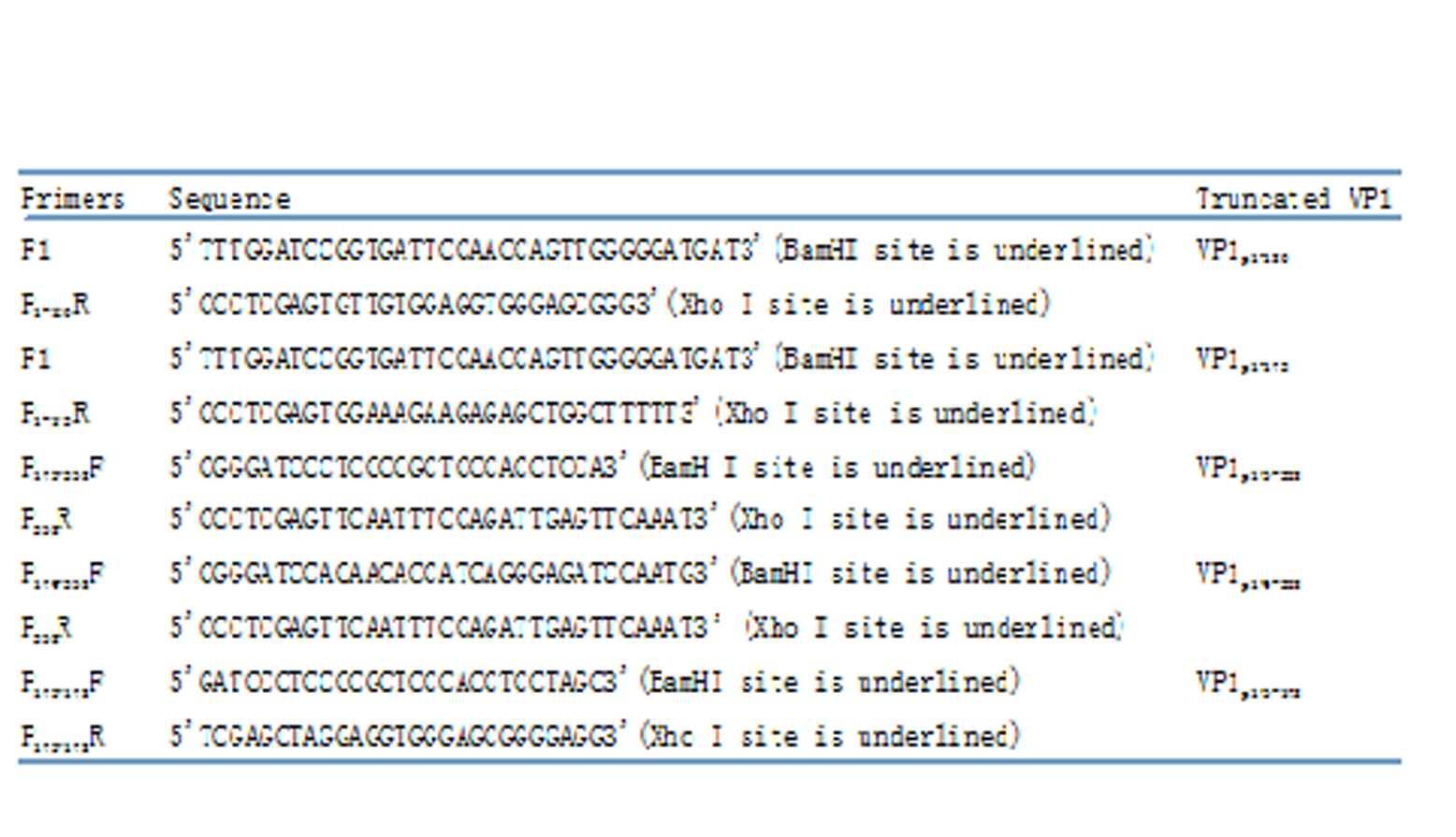

Supplement: S1 Table — (TIF) [file pone.0118041.s001.tif]

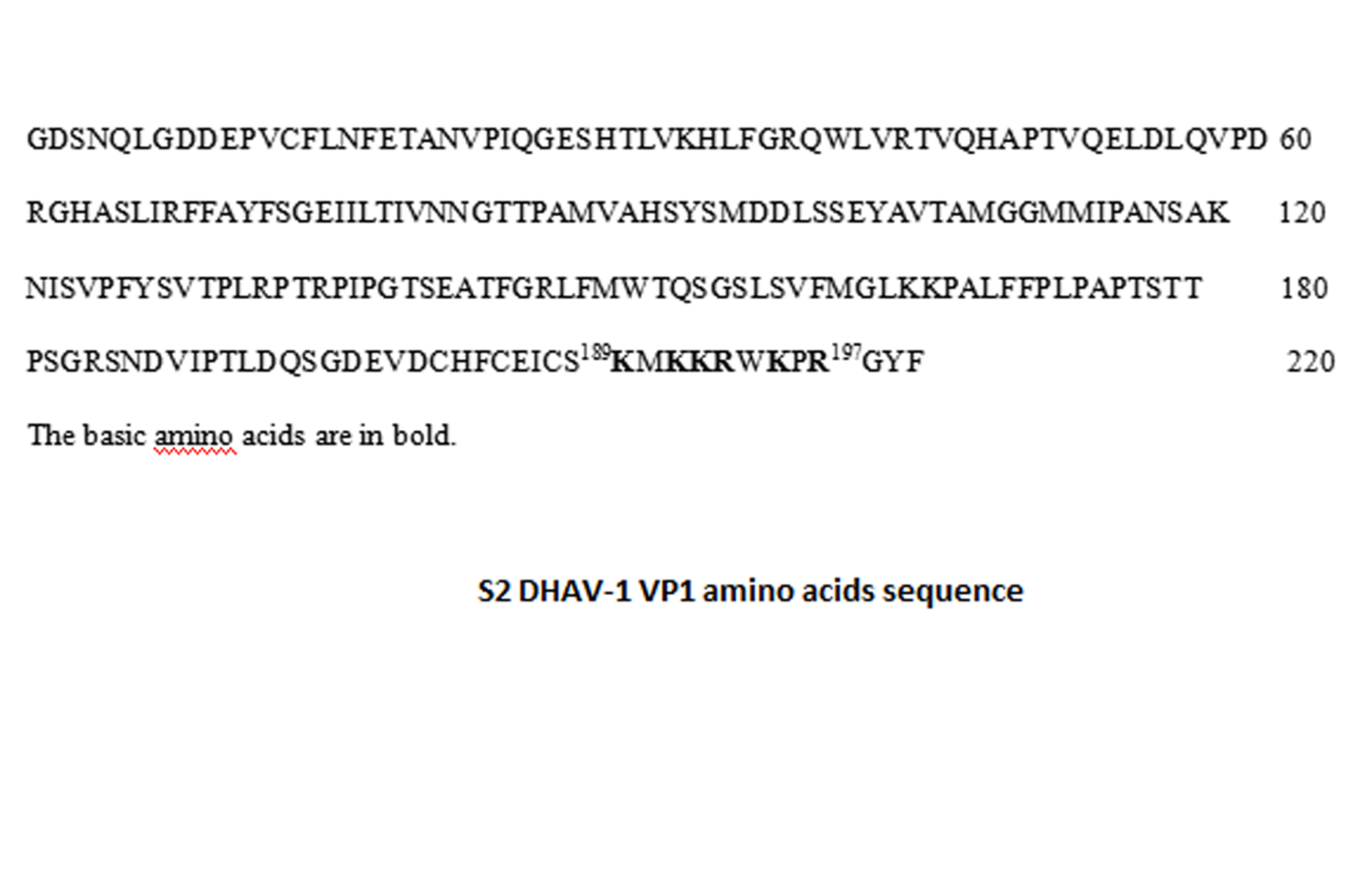

Supplement: S2 Table — (TIF) [file pone.0118041.s002.tif]
